# *Trypanosoma brucei* Lipophosphoglycan Induces the Formation of Neutrophil Extracellular Traps and Reactive Oxygen Species Burst *via* Toll-Like Receptor 2, Toll-Like Receptor 4, and c-Jun N-Terminal Kinase Activation

**DOI:** 10.3389/fmicb.2021.713531

**Published:** 2021-07-28

**Authors:** Kai Zhang, Ning Jiang, Xiaoyu Sang, Ying Feng, Ran Chen, Qijun Chen

**Affiliations:** ^1^Key Laboratory of Livestock Infectious Diseases in Northeast China, Key Laboratory of Zoonosis, Ministry of Education, College of Animal Science and Veterinary Medicine, Shenyang Agricultural University, Shenyang, China; ^2^The Research Unit for Pathogenic Mechanisms of Zoonotic Parasites, Chinese Academy of Medical Sciences, Shenyang, China

**Keywords:** *Trypanosoma brucei*, neutrophil, lipophosphoglycan, neutrophil extracellular trap, c-Jun N-terminal kinase, toll-like receptor, reactive oxygen species

## Abstract

*Trypanosoma brucei brucei* is the causative agent of African animal trypanosomosis, which mainly parasitizes the blood of the host. Lipophosphoglycan (LPG), a polymer anchored to the surface of the parasites, activates the host immune response. In this study, we revealed that *T. brucei* LPG stimulated neutrophils to form neutrophil extracellular traps (NETs) and release the reactive oxygen species (ROS). We further analyzed the involvement of toll-like receptor 2 (TLR2) and toll-like receptor 4 (TLR4) and explored the activation of signaling pathway enzymes in response to LPG stimulation. During the stimulation of neutrophils by LPG, the blockade using anti-TLR2 and anti-TLR4 antibodies reduced the phosphorylation of c-Jun N-terminal kinase (JNK), the release of DNA from the NETs, and the burst of ROS. Moreover, the addition of JNK inhibitor and nicotinamide adenine dinucleotide phosphate (NADPH) oxidase inhibitor exhibited similar effects. Our data suggest that *T. brucei* LPG activates the phosphorylation of JNK through TLR2 and TLR4 recognition, which causes the formation of NETs and the burst of ROS.

## Introduction

*Trypanosoma brucei brucei*, a subspecies of the genus *T. brucei*, infects a variety of animals in Africa and causes widespread epidemics of African animal trypanosomosis (also known as trypanosomiasis or Nagana; Guerrini and Bouyer, 2007; Latif et al., 2019; Odeniran et al., 2020). African animal trypanosomiasis is transmitted by tsetse flies into the hosts during blood-sucking and the infected animals suffer from wasting, anemia, neurological symptoms, and even death (Osaer et al., 1999; Diarra et al., 2019). Other pathogens of this genus can cause sleeping sickness in humans (Kennedy, 2013; Echodu et al., 2015; Buscher et al., 2017); thus, *Trypanosoma*-related diseases cause serious disturbances to agricultural production and people’s lives in Africa.

*Trypanosoma brucei* displays a variety of immunogenic substances on its body surface such as variant surface glycoproteins (VSG) and lipophosphoglycan (LPG; Turco, 1992; Urakawa et al., 1997). The former stimulates the hosts to produce specific antibodies against trypanosomes; however, trypanosomes can evade host-specific immune responses by periodically altering VSG (Manna et al., 2014). LPG, first identified on the surface of *Leishmania*, is a highly complex macromolecule consisting of a glycosylphosphatidylinositol (GPI) anchor, a glycan core, phosphoglycan chains (PG), and terminal oligosaccharide caps (Turco, 1992). As an immunogen, LPG of *Leishmania* activates immune cells to produce cytokines, interferes with the pro-inflammatory host cell responses, and stimulates the release of neutrophil extracellular traps (NETs; Guimaraes-Costa et al., 2009; Franco et al., 2012; Rojas-Bernabe et al., 2014). Like *Leishmania* protozoa, trypanosomes also display LPG on their surfaces (Hublart et al., 1988; Singh et al., 1994).

In *Trypanosoma* and *Leishmania* parasites, a variety of molecules have been reported to bind to different pattern recognition receptors (PRRs) on the surface of immune cells and elicit multiple immune responses through intracellular signaling pathways. For instance, the extracellular vesicles of *Trypanosoma cruzi* could elicit the translocation of NF-κB by interacting with toll-like receptor 2 (TLR2), and *T. cruzi* total lipid extract induced liposome formation through TLR2/6. Further, the GPI of *T. cruzi* could also stimulate the release of multiple cytokines *via* TLR2 and toll-like receptor 4 (TLR4; Campos et al., 2001; Oliveira et al., 2004; Bott et al., 2018; Cronemberger-Andrade et al., 2020). In addition, several studies have found that LPG of *Leishmania major* could interact with TLR2, and LPG of *Leishmania braziliensis* could regulate macrophage activation *via* TLR4 (de Veer et al., 2003; Kavoosi et al., 2009; Vieira et al., 2019). Further, more PRRs have been found to involve in responses to parasitic infections. For instance, *T. cruzi* amastigote and *Leishmania* could regulate host immune response through mannose receptors, *T. brucei* DNA and *Leishmania donovani* CpG DNA could interact with host cell TLR9 (Kahn et al., 1995; Khan et al., 2014; Gupta et al., 2015; Lee et al., 2018; Tiwari et al., 2021). However, it is not clear which PRRs the LPG of *T. brucei* could interact with.

Chemotaxis and phagocytosis are the basic mechanisms of neutrophils in response to pathogen invasion (Alsharif et al., 2015). Neutrophils, as the effector cells of innate immunity, are recruited to the site of infection (Hallett and Dewitt, 2007; Cassatella et al., 2019). Since their first discovery in an *Escherichia coli* infection in 2004, NETs have also been reported in thrombosis, rheumatic diseases, and infections with various pathogens (Brinkmann et al., 2004; Sousa-Rocha et al., 2015; Apel et al., 2018; Wolach et al., 2018; Bouchery et al., 2020). However, only about 30% of neutrophils can be triggered by NETs (Grob et al., 2020). NETs are composed of DNA, which forms the skeleton of the network structure, and a variety of proteases [such as histone, elastase, and myeloperoxidase (MPO); Beiter et al., 2006; Papayannopoulos et al., 2010; Neeli and Radic, 2012]. In response to protozoan infection, host neutrophils could generate NETs, while pathogens, such as *Plasmodium falciparum* can secrete deoxyribonucleases, to evade capture by host traps (Chang et al., 2016). Recently, Grob et al. (2020) and we reported that trypanosomes could stimulate the formation of NETs by neutrophils (Zhang et al., 2021). However, the underlying molecular mechanism is unknown. The observation that the structural properties of LPG of *T. brucei* are consistent with those of *Leishmania* (Hublart et al., 1988) inspired us to further investigate the role of trypanosomal LPG in the interaction with host immune cells.

In the present study, LPG of *T. brucei* was extracted and its induction of NETs and reactive oxygen species (ROS) from neutrophils was investigated. It has also been reported that the release of NETs depends on the activation of protein kinases such as p38 mitogen-activated protein kinases (p38 MAPKs) in keratinocyte exosome-induced NETs and c-Jun N-terminal kinase (JNK) in tetrachlorobenzoquinone (TCBQ)-induced NETs (Jiang et al., 2019; Lv et al., 2021). Therefore, we tested several inhibitors of protein kinases and only inhibitors of JNK and nicotinamide adenine dinucleotide phosphate (NADPH) oxidase inhibited the formation of LPG-induced NETs. Our data provide further insight into the mechanism by which *T. brucei* induces the formation of NETs and the burst of ROS from neutrophils.

## Materials and Methods

### Animals

Female BALB/c mice (about 20–22 g body weight) were purchased from Liaoning Chang Sheng Biological Technology Company (Permit No. SYXK<Liao>2020-0001) in China. All animal experiments were performed according to the institutional guidelines on animal welfare and ethical permissions. The study was approved by the Ethical Committee of Shenyang Agricultural University, China (Clearance No. 2015-CAV-01).

### Parasite Culture and Purification

Bloodstream *T. brucei* Lister 427 was cultured *in vitro* in the HMI-11 medium (Zhang et al., 2021) containing 10% heat-inactivated fetal bovine serum (FBS) at 37°C with 5% CO_2_. Bloodstream parasites (5 × 10^7^/ml) from infected mouse blood were purified using a DE52 cellulose column as described previously (Lanham and Godfrey, 1970).

### Neutrophil Extraction

Neutrophils from mouse bone marrow were obtained by density gradient centrifugation according to the protocol of a Mouse Bone Marrow Neutrophil Isolation Kit (Solarbio, Beijing, China; Boyum, 1974; Swamydas and Lionakis, 2013). Briefly, reagents A and C were sequentially added to a 15-ml tube in the ratio of 1:2, and then the cell suspension was dropped onto the surface of the separation liquid. The sample was centrifuged at 800 × *g* for 30 min, then the neutrophil fraction between reagent C and reagent A was removed with a pipette. The neutrophils were washed three times with 10 ml phosphate-buffered saline (PBS) and centrifuged at 250 × *g* for 10 min (Zhang et al., 2021). Purified neutrophils were cultured in Roswell Park Memorial Institute (RPMI) medium 1640 (Sigma, St. Louis, MO, United States) containing 10% FBS at 37°C with 5% CO_2_.

### Lipophosphoglycan Purification

Bloodstream *T. brucei* parasites (1 × 10^10^) purified from the blood of infected mice were harvested and their LPG was extracted and purified as described previously (McConville et al., 1987; Wilson et al., 1999). Briefly, the collected parasites were washed with sodium phosphate buffer and extracted with 20 times the volume of chloroform/methanol/water (4:8:3, *v*/*v*) for 1 h at room temperature with continuous vigorous shaking. Thereafter, the precipitate was collected by centrifugation at 3,500 rpm for 30 min. The precipitate was extracted twice with water-saturated 1-butanol at 4°C, with continuous stirring for 18 h each time, and then the samples were centrifuged at 10,000 × *g* for 30 min to remove the insoluble material. The supernatant was lyophilized and washed using chloroform/methanol (2:1, *v*/*v*), and the residue was purified using graded separation by octyl agarose chromatography with a 1-propanol gradient (5–60%) in 0.1 M 2-[tris(hydroxymethyl)methyl]-amino ethanesulfonate (Tes) buffer.

### Preparation of the Parasite Ghosts

Saponin at 10% was added to the trypanosome culture at a ratio of 1:100 and incubated on ice for 7 min. The precipitate was collected and washed three times using PBS to remove the contents and retain the ghosts of the trypanosomes.

### LPG Analysis Using Liquid Chromatography-Mass Spectrometry

The LPG samples were centrifuged at 13,300 rpm for 10 min, and 50 μl of the supernatant solution was taken for detection. A triple time-of-flight (TOF) 6600 high-resolution mass spectrometer (AB SCIEX, Coppell, TX, United States) with an electrospray negative ion source was used, with the following mass spectrometry parameter settings: TOFMS scan mode; declustering potential (DP), −30 V; collision energy (CE), −5 eV; ion source temperature, 400°C; spray voltage, 4,500 V; and Curtain Gas. The injection volume was 1 μl of mixed mobile phase (0.05 ml/min) for mass spectrometry analysis.

### Localization of *T. brucei* LPG Using an Indirect Immunofluorescence Assay

Parasites or ghosts (2 × 10^5^) were coated onto slides and fixed with cold methanol for 15 s at room temperature, followed by blocking with 3% bovine serum albumin at 37°C for 30 min. The samples were incubated with mouse monoclonal anti-LPG IgM antibodies (1:500; Cedarlane, Burlington, ON, Canada) and healthy mouse IgM was used as the control. The samples were then stained with fluorescein isothiocyanate (FITC)-conjugated goat anti-mouse IgM (1:200; Thermo Fisher Scientific, Waltham, MA, United States) for 1 h at 37°C. DNA was stained using diamidino-2-phenylindole (DAPI) before the images were captured under a confocal microscope (Leica Camera AG, Wetzlar, Germany).

### LPG Detection Using Western Blotting

Following the method detailed in a previous study (Castro-Sesquen et al., 2014), the 10 μg purified LPG or *T. brucei* total lysate was mixed with 1 × sodium dodecyl sulfate polyacrylamide-gel electrophoresis (SDS-PAGE) loading buffer and then heated at 100°C for 5 min. The samples were separated on 10% Tris-Glycine polyacrylamide gels and transferred onto polyvinylidene difluoride membranes (PVDF; Millipore, Billerica, MA, United States) using a Mini Trans-blot system (Bio-Rad, Hercules, CA, United States). After being blocked with 5% (*w*/*v*) skim milk in PBS for 1 h at 37°C, the membranes were incubated overnight with mouse monoclonal anti-LPG IgM (1:1,000; Cedarlane) and healthy mouse IgM as a control. After washing with PBS-Tween20 (PBST), the membranes were incubated with horseradish peroxidase (HRP)-conjugated goat anti-mouse IgM (1:1,000; Sangon Biotech, Shanghai, China) for 1 h at 37°C. Finally, the samples were visualized using electro-chemiluminescence (Solarbio).

### Quantification of DNA Released From NETs After LPG Stimulation

Isolated mouse neutrophils were added into 24-well plates (1 × 10^6^ per well) and NET formation was induced for 3 h using 100 nM phorbol-12-myristate-13-acetate (PMA), 50 μg/ml lipopolysaccharide (LPS), 50 μg/ml *T. brucei* total lysate, or 50 μg/ml *T. brucei* LPG, respectively. Thereafter, restriction enzymes (*EcoR* I and *Hind* III, 20 U/ml; Takara, Otsu, Japan) were added to the cells, which were further incubated for 2 h at 37°C. NET-DNA was quantified in the supernatant using a PicoGreen double-stranded DNA (dsDNA) kit (Invitrogen, Carlsbad, CA, United States) and analyzed by a fluorescent enzyme marker (PerkinElmer, VICTOR Nivo, Waltham, MA, United States). To explore the effect of LPG concentration and time on the NET formation, LPG (25, 50, 100, 200, or 300 μg/ml) was co-incubated with neutrophils for 3 h, or 50 μg/ml LPG was co-incubated with neutrophils for 30, 60, 120, or 180 min. To explore the effect of the enzymes in the signaling pathway on the formation of NETs, 20 μM diphenyleneiodonium chloride (DPI, an NADPH oxidase inhibitor; Selleck, Houston, TX, United States), 10 μM SP600125 (JNK inhibitor; Selleck), 10 μM 4-aminobenzohydrazide (myeloperoxidase inhibitor; Sigma), 10 μM R0318220 [protein kinase C (PKC) inhibitor; Selleck], 20 μM PD98059 [extracellular regulated protein kinases (ERK) inhibitor; Selleck], or 40 μM SB203580 (p38 MAPK inhibitor; Selleck) were, respectively, added and incubated with the cells for 30 min, 2 h, 2.5 h, 30 min, 2 h, and 2 h before addition of 50 μg/ml LPG, as described previously (Rojas-Bernabe et al., 2014; Khan et al., 2017; Mendez et al., 2018; Mohanty et al., 2019).

### Role of Neutrophil TLR2 and TLR4 in NET Release Induced by *T. brucei* LPG

Neutrophils (1 × 10^6^) were co-incubated with the polyclonal anti-TLR2 or/and anti-TLR4 antibodies (20 μg/ml; CLOUD-CLONE CORP, Wuhan, Hubei, China) at 37°C for 1 h before stimulation with 50 μg/ml *T. brucei* LPG for 3 h at 37°C. The isotypes of IgG were used in all control wells. Released NET-DNA was detected as described in the previous section. The effectiveness of blocking TLR2 and TLR4 receptors was assessed by measuring chemokine IL-8 in the supernatant of neutrophils stimulated with Pam2CSK4 (10 ng/ml; Selleck) and LPS (50 ng/ml; Sigma), respectively (Acorci-Valerio et al., 2010; Noh et al., 2015). IL-8 was measured using an enzyme-linked immunosorbent assay (ELISA) kit (MEIMIAN, Wuhan, Hubei, China).

### Detection TatD DNases in Ghosts by Indirect Immunofluorescence

Saponin-treated or untreated *T. brucei* (5 × 10^5^) were spread on glass coverslips treated with 0.01% polylysine, followed by blocking with 3% bovine serum albumin at 37°C for 30 min. The samples were incubated with rat anti-TatD05-specific serum (1:100) and rabbit anti-TatD15-specific serum (1:100) for 1 h at 37°C and then stained with Alexa Fluor 594-conjugated goat anti-rat IgG (1:600; Thermo Fisher Scientific) and Alexa Fluor 488-conjugated goat anti-rabbit IgG (1:600; Thermo Fisher Scientific) for 1 h at 37°C. The DNA was stained with DAPI before images were captured under a confocal microscope.

### Detection of NETs Induced by Parasite Ghosts and LPG in Indirect Immunofluorescence

A total of 1 × 10^5^ neutrophils were spread on glass coverslips treated with 0.01% polylysine, which were allowed to co-incubate with either *T. brucei* (5 × 10^5^), *T. brucei* ghosts (5 × 10^5^) or were left unstimulated for 3 h at 37°C. Followed by blocking with 3% bovine serum albumin at 37°C for 30 min, the samples were incubated with rat anti-Elastase (1:1,000; Thermo Fisher Scientific) and rabbit anti-Histone (1:1,000; Thermo Fisher Scientific). After three washes, the samples were stained with Alexa Fluor 594-conjugated goat anti-rat IgG (1:600; Thermo Fisher Scientific) and Alexa Fluor 488-conjugated goat anti-rabbit IgG (1:600; Thermo Fisher Scientific) for 1 h at 37°C. The DNA was stained with DAPI before images were captured under a confocal microscope.

A total of 1 × 10^5^ neutrophils were spread on glass coverslips treated with 0.01% polylysine, which were allowed to incubate with *T. brucei* LPG (50 μg/ml) or untreated for 3 h at 37°C. Followed by blocking with 3% bovine serum albumin at 37°C for 30 min, the samples were incubated with rat anti-Elastase (1:1,000; Thermo Fisher Scientific) and rabbit anti-Histone (1:1,000; Thermo Fisher Scientific). After three washes, the samples were stained with Alexa Fluor 594-conjugated goat anti-rat IgG (1:600; Thermo Fisher Scientific) and Alexa Fluor 488-conjugated goat anti-rabbit IgG (1:600; Thermo Fisher Scientific) for 1 h at 37°C. The DNA was stained with DAPI before images were captured under a confocal microscope.

### Analysis of the Origin of the DNA in LPG-Induced NETs

Neutrophils (1 × 10^7^) were stimulated with 50 μg/ml *T. brucei* LPG for 3 h and the supernatants were concentrated for PCR analysis. Supernatants without treatment, neutrophil mitochondrial DNA, and nuclear DNA were used as controls. All the samples were assayed by amplifying three nuclear and four mitochondrial genes (Yousefi et al., 2008). The primer sequences of the genes were as follows: *Atp6* (encoding ATP synthase subunit 6; 5ꞌ-CTCCATTCTTTCCAACACTGACT-3ꞌ and 5ꞌ-TTAATACTAGAGTAGCTCCTCCGAT-3ꞌ), *Co1* (encoding cytochrome oxidase c subunit 1; 5ꞌ-AACTCATCCCTTGACATCGTGC-3ꞌ and 5ꞌ-GGCAGCCATGAAGTCATTCTAAA-3ꞌ), *Nd1* (encoding NADH dehydrogenase subunit 1; 5ꞌ-ACATTGTTGGTCCATACGGCA-3ꞌ and 5ꞌ-GGTCAGGCTGGCAGAAGTAATC-3ꞌ), *Cytb* (encoding cytochrome oxidase b; 5ꞌ-CTTGACCCGATTCTTCGCTTT-3ꞌ and 5ꞌ-TAGGCTTCGTTGCTTTGAGGTAT-3ꞌ), *Gapdh* (encoding glyceraldehyde-3-phosphate dehydrogenase; 5ꞌ-GCGAGACCCCACTAACATCAAA-3ꞌ and 5ꞌ-TCTCCCCACTGCCTACATACCA-3ꞌ), *Rhoh* (encoding Ras homolog family member H; 5ꞌ-TTCACCTCTGAGACCTTCCCG-3ꞌ and 5ꞌ-AGGGCTGAGCACTCCAGGTAG-3ꞌ), and *Actb* (encoding beta-tubulin; 5ꞌ-TCCCTGTATGCCTCTGGTCGTA-3ꞌ and 5ꞌ-AACTTACCCAAGAAGGAAGGCTG-3ꞌ).

DNA (100 ng) was amplified using primers at 1 μM. The cycling parameters were as follows: an initial 94°C for 2 min; then 32 cycles of 94°C for 30 s, 58°C for 30 s, and 72°C for 1 min; and then 72°C for 10 min. The PCR products were separated using 4% agarose gel and stained with ethidium bromide.

### Co-immunoprecipitation and Immunoblotting

A total of 5 × 10^6^ neutrophils were separately incubated with 10 mg/ml of bloodstream *T. brucei* LPG or RPMI alone for 1 h at 37°C, 5% CO_2_. Cells were washed and lysed in 250 μl radioimmunoprecipitation (RIPA) lysis buffer (Beyotime Biotechnology, Shanghai, China). Five hundred microliter of 1 mg/ml supernatant was incubated with 20 μl anti-TLR2 or 20 μl anti-TLR4 overnight at 4°C with shaking. The immunocomplexes were captured with protein A-agarose beads (General Electric Company, Fairfield, CT, United States) for 2 h on ice under constant mild agitation. The beads were washed five times in cold washing buffer (0.05 M Tris, pH 7.4, 150 mM NaCl, 10 mM EDTA, and 1% NP-40), centrifuged at 14,000 × *g* for 10 min at 4°C, which were resuspended with 80 μl PBS and add loading buffer, and boiled for 10 min. Samples were centrifuged at 14,000 × *g* for 10 min at 4°C, and the supernatant was transferred to a new tube.

The samples were dissolved on 10% Tris-Glycine polyacrylamide gels and transferred onto PVDF using a Mini Trans-blot system. After being blocked with 5% (*w*/*v*) skim milk in PBS for 1 h at 37°C, the membranes were incubated overnight with the mouse monoclonal anti-LPG IgM (1:1,000; Cedarlane). After washing with PBST, the membranes were incubated with HRP-conjugated goat anti-mouse IgM (1:1,000; Sangon Biotech) for 1 h at 37°C. Finally, the signals were visualized using electrochemiluminescence (Solarbio).

### Co-localization of TLR and LPG on the Surface of Neutrophils by Indirect Immunofluorescence

Neutrophils (1 × 10^5^) were seeded onto poly-Lysine treated glass coverslips. Then, 50 μg/ml of *T. brucei* LPG was added to the cells, which were co-incubated at 37°C for 3 h. The samples were fixed using 4% paraformaldehyde for 30 min at room temperature, followed by blocking with 3% bovine serum albumin at 37°C for 30 min. Rabbit anti-TLR2 (1:100; CLOUD-CLONE CORP) or rabbit anti-TLR4 (1:100; CLOUD-CLONE CORP) antibodies and mouse anti-LPG IgM (1:500; Sangon Biotech) were incubated with the samples for 1 h at 37°C, and the healthy mouse IgM and rabbit IgG were used as negative controls. Then the samples were, respectively, stained with FITC-conjugated goat anti-mouse IgM (1:200; Sangon Biotech) and Alexa Fluor 594-conjugated goat anti-rabbit IgG (1:600; Thermo Fisher Scientific) for 1 h at 37°C (Becker et al., 2003), and then the fluorescence signal was recorded with a confocal laser scanning microscope (Leica SP8).

### Analysis of JNK Phosphorylation During LPG-Induced NET Production

Neutrophils (1 × 10^6^) were co-incubated with polyclonal anti-TLR2 and/or anti-TLR4 (20 μg/ml) antibodies (CLOUD-CLONE CORP) at 37°C for 1 h before stimulation with 50 μg/ml *T. brucei* LPG for 3 h at 37°C. In parallel, 10 μM SP600125 was added to inhibit JNK phosphorylation for 2 h before LPG stimulation of the cells. Additionally, a concentration gradient of LPG (0, 25, 50, and 100 μg/ml) was added to the neutrophil culture wells. Cells from each well were collected, washed, and broken, and supernatants were collected for western blotting analysis using primary antibodies comprising rabbit monoclonal anti-phosphorylated JNK and anti-JNK (1:1,000; Beyotime Biotechnology), and the secondary antibody was HRP-conjugated goat anti-rabbit IgG (1:25,000; ZSGB-BIO, Beijing, China).

### Analysis of Oxidative Burst After LPG Stimulation

Neutrophils were co-incubated with 50 μg/ml *T. brucei* LPG and cellular ROS production was detected at different time points according to product specifications of the Cellular Reactive Oxygen Species Assay Kit (Beyotime Biotechnology). In parallel, 20 μM DPI, 10 μM SP600125, 20 μM PD98059, 40 μM SB203580, and polyclonal anti-TLR2 and anti-TLR4 antibodies were preincubated separately with the neutrophils at 37°C for 30 min, 2 h, 2.5 h, 30 min, 2 h, and 2 h, respectively. Thereafter, 50 μg/ml *T. brucei* LPG was added to the wells and incubated with cells at 37°C for 30 min, and the ROS in the supernatant was detected.

### Statistical Analyses

All data were analyzed using GraphPad Prism 5.0 (GraphPad Software, Inc., La Jolla, CA, United States) and PASW Statistics 18 (IBM Corp., Armonk, NY, United States). Two groups of data were compared using a *t*-test, while multiple groups of data were compared using one-way analysis of variance (ANOVA). Means and standard deviations (SD) were determined using three biological replicates; *p* < 0.05 was considered as significant and *p* < 0.01 was considered as highly significant.

## Results

### Localization and Extraction of *T. brucei* LPG

**Figure 1:**
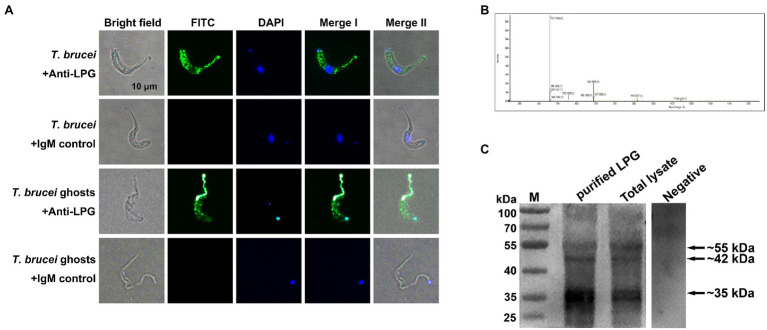
Localization and characterization of *Trypanosoma brucei* lipophosphoglycan (LPG). (**A**; *n* = 4) Detection of *T. brucei* LPG by indirect immunofluorescence. Mouse anti-LPG IgM monoclonal antibodies were used as the primary antibodies. Healthy mouse IgM was used as the negative control. Fluorescein isothiocyanate (FITC)-conjugated goat anti-mouse IgM was used as the secondary antibody. Nucleic acids and kinetoplasts were stained blue using diamidino-2-phenylindole (DAPI), meanwhile, the LPG was stained green. Scale bar: 10 μm. (**B**; *n* = 5) Detection of the mass-to-charge ratio (*m*/*z*) of *T. brucei* LPG after purification using LC-MS. (**C**; *n* = 3) LPG in extracts and the lysates of parasites were analyzed using western blotting. Ten microgram purified LPG or *T. brucei* total lysate was used as the sample. Mouse monoclonal anti-LPG IgM was used as the primary antibody. Negative: 10 μg purified LPG was used as the sample and the healthy mouse IgM was used as the primary antibody. Horseradish peroxidase (HRP)-coupled goat anti-mouse IgM antibody was used as the secondary antibody.

Through indirect immunofluorescence analysis, LPG has been detected on both the surface of *T. brucei* and on the parasite ghosts (Figure 1A). The liquid chromatography-mass spectrometry (LC-MS) detection of the mass-to-charge ratio (*m*/*z*) peak plots showed that we extracted a polymer with a spacing of 229 between the four main peaks (457, 686, 915, and 1,144), indicating the presence of a structural unit with an *m*/*z* ratio of 229 in the *T. brucei* LPG structure (Figure 1B). We detected LPG in *T. brucei* extracts and total lysates using western blotting with specific monoclonal antibodies recognizing LPG. Target bands were detected with the sizes of approximately 55, 42, and 35 kDa, respectively, which corresponded with those mentioned in the manual for the anti-LPG monoclonal antibody (Figure 1C).

### LPG on the Surface of *T. brucei* Could Stimulate the Formation of NETs

**Figure 2:**
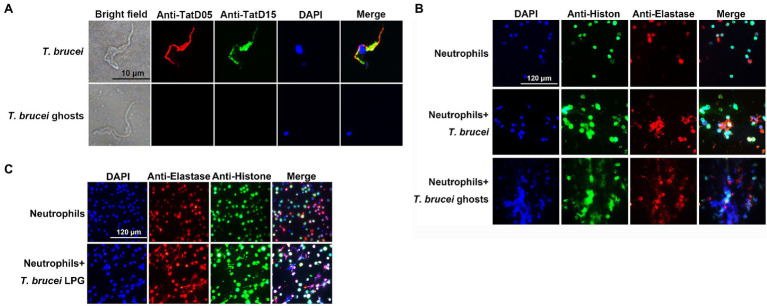
The formation of neutrophil extracellular traps (NETs) was detected using an indirect immunofluorescence assay (IFA). (**A**; *n* = 3) The visualization of TatD DNases in *T. brucei* after saponin treatment. Rat anti-TryTatD05 and rabbit anti-TryTatD15 serum were used as the primary antibodies. Alexa Fluor 594-conjugated goat anti-rat IgG and Alexa Fluor 488-conjugated goat anti-rabbit IgG were used as the secondary antibodies. Nucleic acids and kinetoplasts were stained blue using DAPI. Meanwhile, TryTatD05 was stained red and TryTatD15 was stained green. Scale bar: 10 μm. (**B,C**; *n* = 3) The generation of ghosts- or LPG-induced NETs was analyzed using an IFA. Rabbit anti-Histone and rat anti-Elastase IgG were used as the primary antibodies. The secondary antibodies were the same as in **(A)**. Nucleic acids were stained blue with DAPI. Histone and elastase were stained green and red, respectively. Scale bar: 20 μm.

*Trypanosoma brucei* could secret TatD deoxyribonuclease, which hydrolyzed NETs (Zhang et al., 2021). However, the trypanosome ghosts pretreated with saponin no longer contained this enzyme and the nuclei were released from the parasites (Figure 2A). Indirect immunofluorescence showed that both *T. brucei* ghosts and the extracted LPG cold stimulated neutrophils to generate NETs (Figures 2B,C).

### Characterization of NET Formation After LPG Stimulation

**Figure 3:**
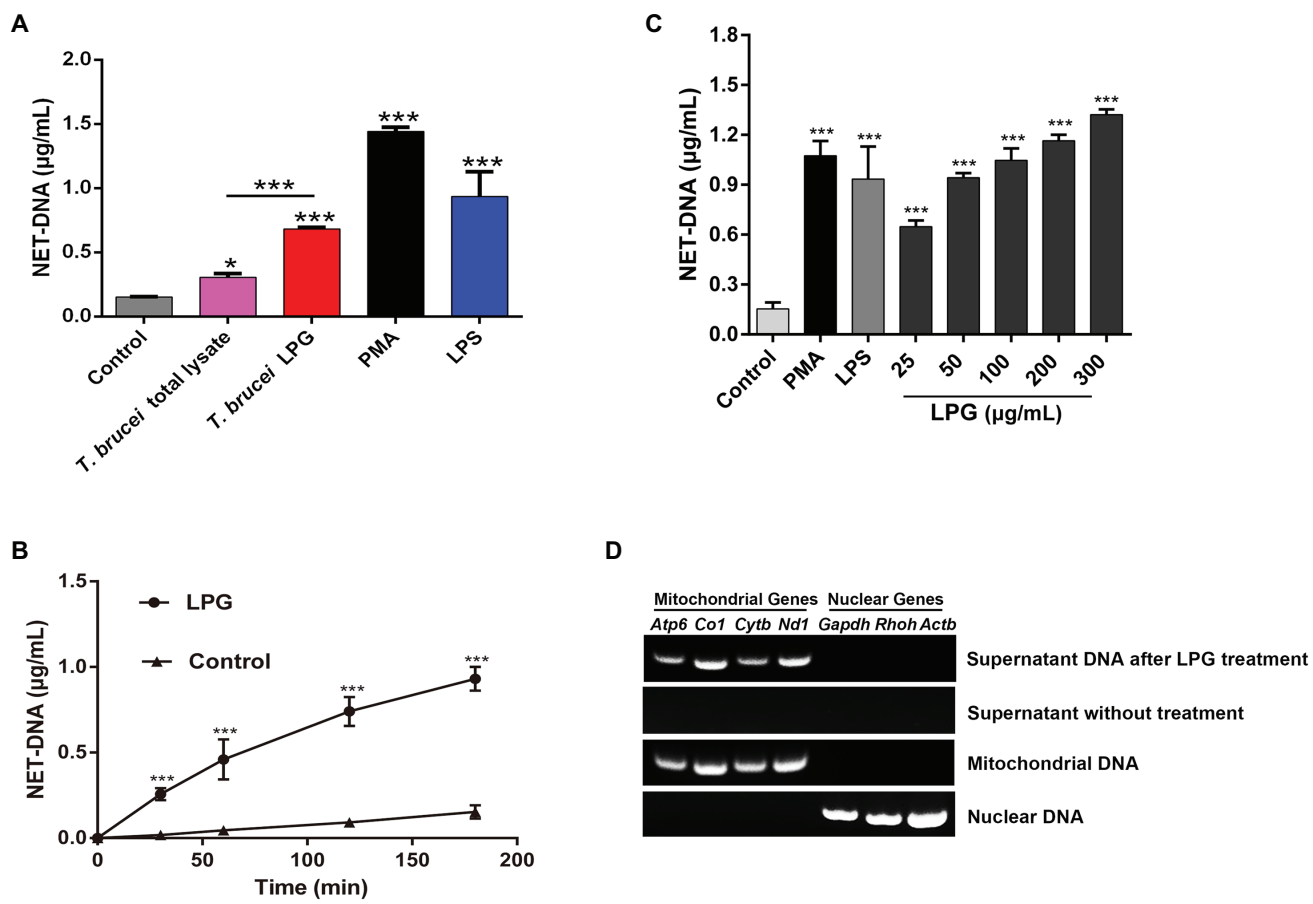
Characterization of LPG-induced NETs. (**A**; *n* = 3) Detection of the DNA content of NETs induced by stimuli. Neutrophils (1 × 10^6^) were, respectively, stimulated for 3 h by 100 nM phorbol-12-myristate-13-acetate (PMA), 50 μg/ml lipopolysaccharide (LPS), 50 μg/ml *T. brucei* total lysate, or 50 μg/ml *T. brucei* purified LPG. NET-DNA was analyzed by fluorescence zymography. Untreated neutrophils were used as the control group. (**B**; *n* = 3) The exploration of LPG-induced release of NET-DNA at different time points. Fifty microgram per milliliter LPG was co-incubated with neutrophils for 30, 60, 120, or 180 min and NET-DNA was measured by fluorescence enzyme labeler. Untreated neutrophils were used as control. (**C**; *n* = 3) Exploration of NET-DNA released from neutrophils induced by different concentrations of LPG. LPG at 25, 50, 100, 200, and 300 μg/ml were co-incubated with neutrophils at 37°C for 3 h. Untreated neutrophils were used as negative control and neutrophils co-incubated with 100 nM PMA or 50 μg/ml LPS was used as the positive control. (**D**; *n* = 3) The origin of DNA in the NETs was analyzed using PCR. Mitochondrial genes (*Atp6*, *Co1*, *Nd1*, and *Cytb*) and nuclear genes (*Gapdh*, *Rhoh*, and *Actb*) were detected in the supernatant after purified LPG treatment for 3 h or untreated. Mitochondrial DNA and nuclear DNA were used as controls. All the error bars represent the mean ± SD. ^*^p < 0.05 and ^***^p < 0.001.

The DNA content of the supernatant after stimulation by *T. brucei* LPG was significantly higher than that of the negative control, while the amount of NET-DNA detected after stimulation by *T. brucei* lysates was lower than that after stimulation by *T. brucei* LPG (Figure 3A). The DNA content stimulated by LPG increased in a time-dependent manner (Figures 3B,C). We attempted to detect mitochondrial genes (*Atp6*, *Co1*, *Nd1*, and *Cytb*) and nuclear genes (*Gapdh*, *Rhoh*, and *Actb*) in the supernatant of the LPG treatment group and no-LPG treatment group. These genes were not detected in the no-LPG group, while several mitochondrial genes were detected in the LPG group (Figure 3D).

### LPG-Induced Formation of NETs *via* TLR2 and TLR4

**Figure 4:**
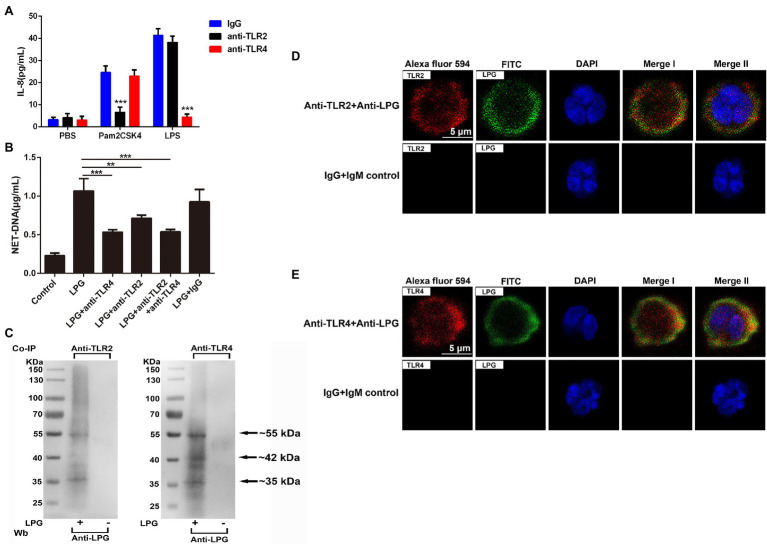
Toll-like receptor 2 (TLR2) and toll-like receptor 4 (TLR4) are involved in the formation of LPG-induced NETs. (**A**; *n* = 3) The effect of TLR2 and TLR4 blockade on the neutrophil surface was analyzed using enzyme-linked immunosorbent assay (ELISA) for IL-8. Twenty microgram per milliliter anti-TLR2, anti-TLR4, and IgG were co-incubated with neutrophils at 37°C for 1 h before the stimulation of cells by Pam2CSK4, LPS, and phosphate-buffered saline (PBS), respectively. IL-8 was detected with an ELISA kit. (**B**; *n* = 3) The role of TLR2 and TLR4 blockade on the LPG-induced NET-DNA content. Twenty microgram per milliliter IgG, 20 μg/ml anti-TLR2, or/and anti-TLR4 were co-incubated with neutrophils at 37°C for 1 h before the stimulation of cells by 50 μg/ml purified LPG. NET-DNA was stained with PicoGreen and analyzed by fluorescence enzyme marker. Untreated neutrophils were used as control. (**C**; *n* = 3) Western blotting analysis of immunoprecipitates. Co-immunoprecipitations of cell lysates of LPG-stimulated or unstimulated neutrophils were performed with TLR2- and TLR4-specific antibodies. Precipitates were subjected to western blotting and probed with anti-LPG. Neutrophils without LPG treatment were used as a negative control. (**D,E**; *n* = 4) The co-localization of TLR2 or TLR4 with LPG using IFA. Rabbit anti-TLR2/TLR4 IgG and mouse anti-LPG IgM were used as the primary antibodies. Alexa Fluor 594-conjugated goat anti-rabbit IgG and FITC-conjugated goat anti-mouse IgM were used as the secondary antibodies. Healthy mouse IgM and rabbit IgG were used as controls. Nucleic acid was stained blue using DAPI, meanwhile, TLR2/TLR4 were stained red and LPG was stained green. TLR2/4 overlap with LPG was indicated in yellow. Scale bar: 5 μm. Error bars for all values represent the mean ± SD. ^**^p < 0.01 and ^***^p < 0.001.

In the blocking assay with TLR-specific antibodies, we observed that 20 μg/ml of anti-TLR2 antibodies significantly inhibited the generation of IL-8 in neutrophils compared to that with Pam2CSK4 (a synthetic diacylated lipopeptide), and incubation with the same concentration of anti-TLR4 antibodies could also significantly reduce the production of IL-8 in LPS-treated neutrophils (Figure 4A). The blockade using the same concentration of the antibodies also reduced the amount of NET-DNA in the supernatant (Figure 4B).

In the immunoprecipitation assays, LPG could be pulled down by both anti-TLR2 and anti-TLR4 antibodies from the neutrophil lysates after incubation, which confirmed that LPG could interact with both TLR2/TLR4 (Figure 4C). The results of indirect immunofluorescence experiments showed that the location of LPG overlapped with that of TLR2 and TLR4 (Figures 4D,E), further suggesting the involvement of the two TLRs in the pathway of LPG-induced NET formation.

### Inhibitors of JNK and NADPH Oxidase Decreased the Generation of NETs Induced by *T. brucei* LPG

**Figure 5:**
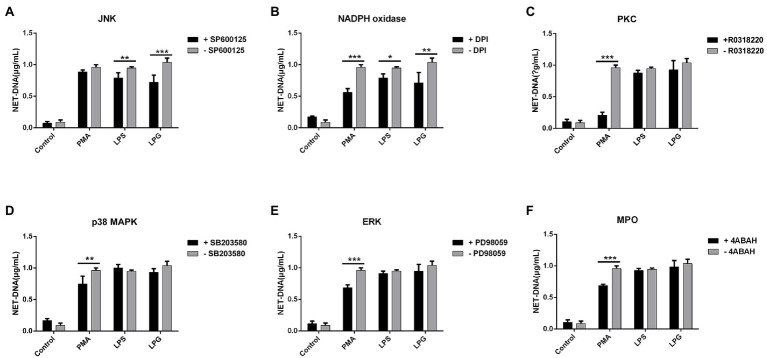
Effect of inhibitors of several protein kinases on NETs formation induced by LPG. (**A-F**; *n* = 3) Ten micromolar SP600125 [c-Jun N-terminal kinase (JNK) inhibitor], 20 μM diphenyleneiodonium chloride [DPI, an nicotinamide adenine dinucleotide phosphate (NADPH) oxidase inhibitor], 10 μM R0318220 [protein kinase C (PKC) inhibitor], 40 μM SB203580 [p38 mitogen-activated protein kinases (p38 MAPK inhibitor)], 20 μM PD98059 [extracellular regulated protein kinases (ERK) inhibitor], and 10 μM 4-aminobenzohydrazide (myeloperoxidase inhibitor) were incubated with neutrophils for 2 h, 30 min, 2 h, 2.5 h, 30 min, 2 h, and 2 h, respectively, before the addition of stimulants (100 nM PMA, 50 μg/ml LPS, and 50 μg/ml purified LPG). Untreated neutrophils were used as control. NET-DNA was represented by fluorescence density. Error bars for all values represent the mean ± SD. ^*^p < 0.05, ^**^p < 0.01, and ^***^p < 0.001.

Both JNK and NADPH oxidase inhibitors could reduce the NET formation after LPS and LPG stimulation (Figures 5A,B), while all the inhibitors of NADPH oxidase, PKC, ERK, p38 MAPK, and MPO significantly inhibited the formation of NETs after PMA treatment (Figures 5B–F).

### TLR2 and TLR4 Signaled the Phosphorylation of JNK After LPG Stimulation

**Figure 6:**
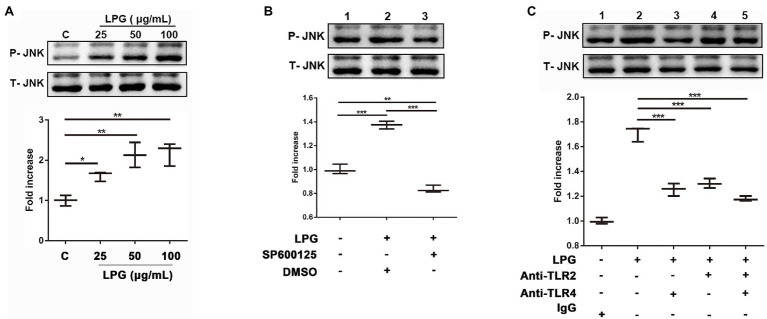
The interaction of LPG with TLR2 and TLR4 induces phosphorylation of JNK. (**A**; *n* = 3) Effect of LPG on JNK phosphorylation. LPG (0, 25, 50, and 100 μg/ml) was added to the neutrophil culture wells, and the supernatants were collected for western blotting analysis using anti-phosphorylated JNK and anti-JNK and the secondary antibody was HRP-conjugated goat anti-rabbit IgG. (**B**; *n* = 3) Effect of JNK inhibitors on JNK phosphorylation. Ten micromolar SP600125 (JNK inhibitor) was incubated with neutrophils at 37°C for 2 h before the addition of 50 μg/ml purified LPG. (**C**; *n* = 3) Effect of TLR2 and TLR4 blockade on JNK phosphorylation following LPG treatment. Neutrophils (1 × 10^6^) were incubated with polyclonal anti-TLR2 and/or anti-TLR4 (20 μg/ml) at 37°C for 1 h before stimulation with 50 μg/ml *T. brucei* LPG for 3 h at 37°C. All data were analyzed by western blotting and grayscale values were analyzed by ImageJ. Error bars for all values represent the mean ± SD. ^*^p < 0.05, ^**^p < 0.01, and ^***^p < 0.001.

Western blotting showed that the levels of phosphorylated JNK were enhanced significantly as the LPG concentration increased (Figure 6A). The JNK inhibitor, SP600125, was able to inhibit the phosphorylation of JNK during LPG-induced NET formation (Figure 6B). However, when we blocked the TLR binding sites on the surface of neutrophils using anti-TLR2 and anti-TLR4 antibodies before LPG stimulation, the level of phosphorylated JNK was reduced (Figure 6C). This implied that LPG could affect JNK phosphorylation *via* TLR2 and TLR4 during NET formation.

### The LPG-Induced ROS Burst Was Dependent on the Activation of NADPH Oxidase and JNK and Was Inhibited by the TLR Blockage

**Figure 7:**
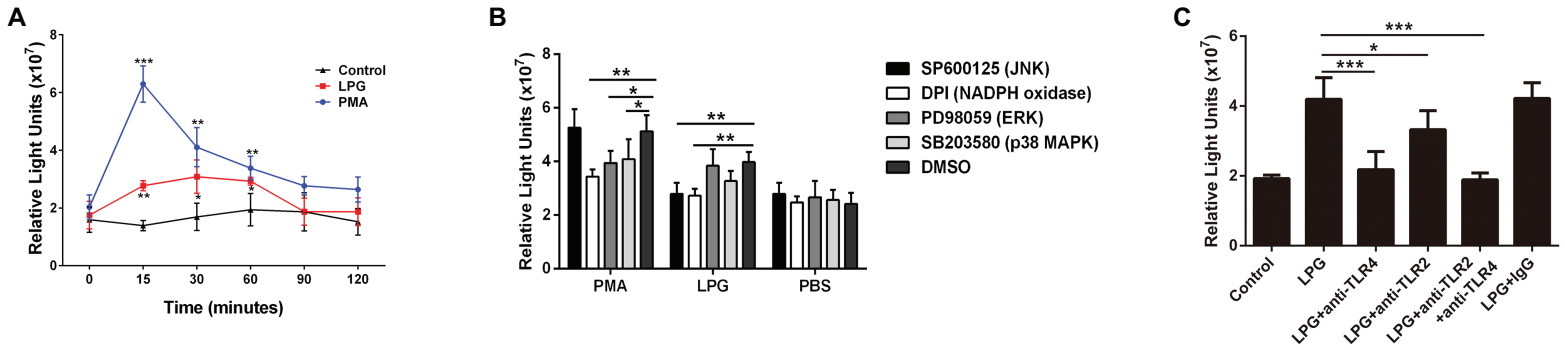
The burst of reactive oxygen species (ROS) induced by *T. brucei* LPG. (**A**; *n* = 3) ROS contents at different time points after LPG or PMA treatment. PMA and PBS treatment were used as positive and negative controls. (**B**; *n* = 3) Analysis of the effect of TLR2 and TLR4 blockade on LPG-induced ROS release. Twenty microgram per milliliter anti-TLR2, anti-TLR4, and IgG were co-incubated with neutrophils at 37°C for 1 h before the stimulation of cells by 50 μg/ml purified LPG. (**C**; *n* = 3) Effect of several signaling pathway enzyme inhibitors on ROS release after LPG treatment. Ten micromolar SP600125 (JNK inhibitor), 20 μM DPI (an NADPH oxidase inhibitor), 40 μM SB203580 (p38 MAPK inhibitor), and 20 μM PD98059 (ERK inhibitor) were co-incubated with neutrophils for 2 h, 30 min, 2.5 h, and 30 min, respectively, before the addition of stimulants (100 nM PMA and 50 μg/ml purified LPG and PBS). DMSO was used as a control. Error bars for all values represent the mean ± SD. ^*^p < 0.05, ^**^p < 0.01, and ^***^p < 0.001.

The ROS assay showed that during the LPG stimulation, a significant increase in ROS levels was detected at 15 min, reaching a maximum at 30 min, and decreasing thereafter. However, the amount of ROS was lower than that induced by PMA (Figure 7A). The p38 MAPK and ERK inhibitors did not affect ROS release, whereas the inhibitors of JNK and NADPH oxidase decreased ROS production (Figure 7B), which suggested that JNK activation was located prior to ROS production. In addition, we investigated the effects of TLR2 and TLR4 on the production of ROS and showed that blockade of TLR2 and TLR4 inhibited the effect of LPG on ROS generation and the inhibitory effect of TLR4 blockade was more pronounced (Figure 7C). These data supported the involvement of TLR2 and TLR4 in the process of LPG-stimulated ROS release.

## Discussion

Neutrophils, the most abundant immune cells in the blood, are generated by the differentiation of myeloid progenitor cells, stored in the bone marrow, and rapidly mobilized into the circulation for action (Liew and Kubes, 2019). Neutrophils move in response to chemotaxis from the blood vessels to the site of infection to phagocytose and break down pathogens (Petri and Sanz, 2018). In addition, antimicrobial peptides and protein enzymes are released by neutrophils to kill pathogens extracellularly (Aarbiou et al., 2006). In the last decade or so, researchers have increasingly identified a new function of neutrophils in many diseases – the production of NETs. NETs contain DNA filaments as a backbone and adhere to histones, elastase, granule proteins, and many other components, which confine the invading pathogens (Papayannopoulos et al., 2010; Thalin et al., 2019). NETs also inhibit the proliferation of pathogens. H1 in the NETs binds and kills certain noroviruses and the fungicidal activity of calmodulin released by NETs was found in a study on *Candida albicans* (Urban et al., 2009; Schonrich and Raftery, 2016). However, it has also been observed that human atopic asthma airways contain a large number of extracellular DNA traps, and mitochondrial DNA deposition has been found in renal biopsy specimens in lupus nephritis and other autoimmune diseases, indicating that NETs are important causative factors involved in autoimmune diseases (Dworski et al., 2011; Mitroulis et al., 2011; Wang et al., 2015; Corsiero et al., 2016; Soderberg and Segelmark, 2016). Furthermore, in the current epidemic of Coronavirus disease 2019 (COVID-19), NETs were found to contribute to organ damage and death (Moschonas and Tselepis, 2019; Barnes et al., 2020; Goel and Kaplan, 2020).

Neutrophil extracellular traps can be induced by various pathways. One of the most studied is the involvement of PMA in regulating the formation of NETs through PKC and Raf-MEK-ERK signaling pathways, as in some gram-negative bacteria, caused by the involvement of non-classical inflammatory vesicles, and in *Staphylococcus aureus* through TLR-signaled formation of non-lytic NETs (Khan et al., 2017; Wan et al., 2017; Chen et al., 2018; Wang et al., 2018). In the indirect immunofluorescence and scanning electron microscopy, we observed that *T. brucei* could stimulate neutrophils to produce NETs as described by Grob et al., 2020 (Zhang et al., 2021); however, the mechanism of NET formation in response to trypanosome was not clear. The ability of *Leishmania* to stimulate NETs release *via* LPG and GPIs has been demonstrated (Wilson et al., 1999), and in *T. cruzi*, blockade of TLR2 and TLR4 receptors inhibited NET production (Sousa-Rocha et al., 2015). Dead trypanosomes could stimulate the release of NETs in our early experiments implied that substances on the parasite surface might be able to stimulate neutrophils to release NETs (Zhang et al., 2021). Since the structural composition of LPG differs in the genus *Leishmania*, it is reasonable to speculate that this difference also exists between *Leishmania* and *Trypanosoma* species (Wilson et al., 1999; Soares et al., 2002, 2004). In the present study, LPG was extracted from the surface of *T. brucei* based on the protocol well-established in studies on *Leishmania* and *Trypanosoma* species (McConville et al., 1987; Hublart et al., 1988; Singh et al., 1994). The purified materials were identified as polymers by LC-MS, which were detected with a LPG-specific antibody in western blotting (Figure 1). In previous studies, lethal *T. brucei* trypomastigotes were able to stimulate neutrophils to produce exocytosis nets (Grob et al., 2020; Zhang et al., 2021). Furthermore, parasite ghosts were also able to stimulate NET formation in this study. This suggests that a substance on the surface of trypanosomes can stimulate the formation of NETs, and this substance was confirmed to be LPG (Figure 2). Indeed, the extracted LPG was confirmed to induce the release of NETs in a time- and concentration-dependent manner (Figures 3A–C).

DNA in NETs can either be released from nuclei or mitochondria depending on the stimulatory substances (Lood et al., 2016; Neubert et al., 2018). Here, several genes from the nucleus and mitochondria were analyzed using PCR, and only the mitochondrial DNA was detected, implying that LPG might primarily stimulate the release of mitochondrial DNA, at least in the initial stage of interaction (Figure 3D).

As previously described, the known forms of trypanosome activator-ligand binding are mainly through toll-like receptors and mannose receptors (Kahn et al., 1995; Khan et al., 2014; Bott et al., 2018; Cronemberger-Andrade et al., 2020). Since LPG of *L. major* and *L. braziliensis* has been reported to interact with either TLR2 or TLR4 (de Veer et al., 2003; Kavoosi et al., 2009; Vieira et al., 2019), we then studied the role of TLR2 and TLR4 in the formation of NETs using anti-TLR2 and anti-TLR4 antibodies to block the binding of LPG to TLR2/4. The effect of antibody blockade was reflected by IL-8 production. Anti-TLR antibodies at 20 μg/ml could block the stimulatory effect of Pam2CSK4 and LPS and inhibit the NET formation after LPG treatment (Figures 4A,B). To further confirm that the NET formation was induced by the interaction of *T. brucei* LPG and TLR2 and TLR4 on the surface of neutrophils, immunoprecipitation using anti-TLR2 and anti-TLR4 antibodies, LPG was specifically precipitated, indicating that *T. brucei* LPG could specifically interact with the two TLRs (Figure 4C). Co-localization of LPG and TLR2/4 was also detected using an immunofluorescence assay (IFA), which further confirmed binding of TLR2 and TLR4 with LPG (Figures 4D,E). These data suggested that *T. brucei* LPG stimulates the release of NETs through TLR2 and TLR4 signally pathways.

There are multiple pathways of intracellular signaling during NET formation, such as the activation of the ERK and p38 MAPK mediates by PMA and Streptococcus Suis Serotype 2 and the activation of the JNK by LPS (Keshari et al., 2013; Khan et al., 2017; Ma et al., 2018). To determine which of the possible pathways are involved in the NET formation, the inhibitors of several signaling enzymes were added to neutrophil cultures before LPG treatment. Only the inhibitors of JNK and NADPH oxidase showed an inhibitory effect on the NET (Figures 5A,B), suggesting that JNK and NADPH oxidase are involved in the induction of NETs by LPG, in a similar manner to that of the LPS-induced NET formation process (Figure 5). The amount of phosphorylated JNK in neutrophils increased after LPG treatment in a concentration-dependent manner, and decreased after blockade with TLR2- and TLR4-specific antibodies, indicating that LPG-TLR interaction primed JNK phosphorylation, which may trigger the activation of NET formation (Figure 6). Furthermore, some studies have shown that respiratory bursts and ROS formation are critical for NETs formation and multiple sources of ROS have been reported to be involved in the formation of NETs, such as NADPH oxidase-derived ROS, singlet oxygen, and HOCl and HOBr (Miyamoto et al., 2006; Nishinaka et al., 2011; Nadesalingam et al., 2018). We and Grob et al. (2020) observed a burst of ROS release during LPG-induced NET formation (Zhang et al., 2021). However, our experiments detected a significant increase in ROS at 15 min, peaking at 30–60 min and then with a gradual decrease, while Grob et al. (2020) detected ROS production after 30 min and was able to maintain it for 2 h. This may be due to the difference in experimental materials, and we used pure LPG while Grob et al. used trypanosomes. Furthermore, the JNK inhibitor and TLR blockade reduced the amount of ROS released, suggesting that LPG-TLR interaction induced the activation of the JNK-mediated pathway prior to ROS production (Figure 7) and possibly NET formation.

In conclusion, *T. brucei* LPG stimulated the release of NETs from neutrophils through the interaction with TLR2 and TLR4, which signaled the JNK-mediated pathway, a process that involves the release of ROS.

## Data Availability Statement

The original contributions presented in the study are included in the article/supplementary material, further inquiries can be directed to the corresponding author.

## Ethics Statement

All animal experiments were performed according to the institutional guidelines on animal welfare and ethical permissions. The study was approved by the Ethical Committee of Shenyang Agricultural University, China (Clearance No. 2015-CAV-01).

## Author Contributions

QC designed and directed the study. KZ performed the experiments. NJ guided the experimental operation and analyzed the data. XS, YF, and RC analyzed the data. QC and KZ wrote the manuscript. All authors contributed to the article and approved the submitted version.

## Conflict of Interest

The authors declare that the research was conducted in the absence of any commercial or financial relationships that could be construed as a potential conflict of interest.

## Publisher’s Note

All claims expressed in this article are solely those of the authors and do not necessarily represent those of their affiliated organizations, or those of the publisher, the editors and the reviewers. Any product that may be evaluated in this article, or claim that may be made by its manufacturer, is not guaranteed or endorsed by the publisher.
